# Longitudinal Analysis of Tactical Strategy in the Men's Division of the Ultimate Fighting Championship

**DOI:** 10.3389/frai.2019.00029

**Published:** 2019-12-17

**Authors:** Lachlan P. James, Alice J. Sweeting, Vincent G. Kelly, Samuel Robertson

**Affiliations:** ^1^Department of Dietetics, Nutrition and Sport, School of Allied Health, La Trobe University, Melbourne, VIC, Australia; ^2^Institute for Health and Sport, Victoria University, Melbourne, VIC, Australia; ^3^School of Exercise and Nutrition Sciences, Queensland University of Technology, Brisbane, QLD, Australia

**Keywords:** mixed martial arts, combat sport, sports analytics, data mining, notational analysis

## Abstract

This study explored longitudinal changes in contemporary mixed martial arts (MMA) combat within the Ultimate Fighting Championship (UFC). A secondary aim was to investigate how bout duration influences the contribution of performance indicators on outcome. Data were acquired via the official analytics provider to the UFC (FightMetric). Male fights with a winner from between 2000 and 2015 (*n* = 2,831) were examined, with 13 common performance indicators attained during each round for each participant along with duration (min) and year of fight. Non-metric dimensional scaling (nMDS) was used to examine bout characteristics by year. The Repeated Incremental Pruning to Produce Error Reduction (RIPPER) algorithm was run to determine a set of rules to explain bout outcome. The nMDS displayed that winning bout performance indicator attributes were dissimilar across the years. Eight rules were generated from the RIPPER, with fight duration featuring in three of eight rules. Distinct shifts occurred (albeit without linear trend) in performance indicator characteristics during the observed period. This was characterized by a more diverse combat style in the years following 2008. However, offensive grappling has remained a key factor regardless of year, and is influenced by bout duration.

## Introduction

Mixed martial arts (MMA) is a combat sport characterized by an amalgamation of techniques from traditional combat sports including wrestling, kick boxing, Brazilian jiu-jitsu, and judo (James et al., 2016). These demands require competitors to possess highly developed technical, physical, and sensorimotor skills. As a result, there are a considerable number of techniques available to the competitor and a broad range of methods for achieving victory. The highest level of professional competition occurs in the Ultimate Fighting Championship (UFC), with the first event held in 1993 (Maher, 2009). The limited rules, which governed the sport in its infancy, allowed drastic changes in the nature of combat patterns (akin to “game-play” in team sports) as fighters evolved from one dimensional representatives from a single combat sport to athletes who employed a greater range of techniques and strategies (Maher, 2009). However, the absence of a thorough rule set in the initial years of the sport (characterized by a limited use of rounds, time limits, weight classes, and judges' scoring) often lead to extended stalls in activity or brief one sided encounters between poorly matched opponents (Gullo, 2013).

As part of a process to legitimize the sport and protect the health of the competitors, the UFC enacted the Unified Rules of MMA in November 2000 (Smith, 2009). In addition to scoring, time- and weight—limit changes, these rules also eliminated a number of techniques including certain strikes (e.g., to the back of the head and neck), and mandated regulatory oversight and medical screening (Maher, 2009). This represented a major turning point in the behavior of MMA combat patterns and formed the framework for the modern iteration of the sport. While physical qualities clearly contribute to success in modern MMA (James et al., 2017a, 2018), these rules changes resulted in an increased technical demand. Athletes no longer represented a single discipline or the sum of distinct techniques from multiple sports, but rather a gestalt with complex technique interactions developed specifically for MMA (Maher, 2009).

In accordance with these changes has come contemporary training environments, that are enhanced in both equipment and scientific knowledge (Birren and Schmitt, 2017). Recent research has demonstrated the considerable influence of skill on outcome in modern MMA. For example, an analysis of performance indicator profiles in the sport revealed that attack attempts had no bearing on outcome (win or loss), whereas the ability to precisely land an attack was a key factor in achieving victory (James et al., 2017b). This suggests that contemporary fighter tactics should focus on the precision of technique, rather than on the capacity to execute a higher volume of attempts that may or may not be successful. Although grappling based combat is the key factor in achieving victory in UFC bouts (James et al., 2017b), recently, athletes (and coaches) appear to have adopted tactics that more effectively mitigate such techniques, allowing for a greater expression of distance striking attacks. Despite this observation, there is an absence of research that objectively describes the longitudinal changes of combat activity within modern-day MMA.

Considering the interest surrounding the evolution of MMA combat, the lack of research into such changes represents a notable knowledge gap. This is despite recent investigations revealing alterations in competition characteristics over several years both within (Franchini et al., 2013; Davis et al., 2018) and outside (Woods et al., 2017a) of combat sports. However, while these previous combat sport investigations provide some insight into the adjustment in tactics as a result of rule changes, they provide little insight into the longitudinal development of combat within MMA, nor do they describe combat changes in the absence of major rule amendments.

A distinctive characteristic of MMA, comparative to many other sports, is the potential for a bout to end at any given moment within the scheduled time. Although this is a common feature of most combat sports, there is a considerably greater number of attacking maneuvers available in MMA, leading to numerous methods and opportunities to achieve victory (and therefore end the bout) prior to completion of the scheduled time. Because of this fluid time-constraint, the relationships between individual performance indicators and outcome have the potential to change as a function of bout duration. However, the nature of the interaction between performance indicators and the duration of a bout has yet to be reported in the literature. It is possible that the profiles generated in earlier studies may relate more to bouts of longer or shorter time spans, which can limit the operationalization of the findings.

The primary objective of this study was to investigate longitudinal changes in contemporary MMA combat patterns via performance indicator attributes of fighters at the highest level of competition (the UFC). Such findings could provide objective information on the development of competition demands and tactical strategies in modern-day MMA. A secondary aim was to investigate how bout duration influences the contribution of specific performance indicators to outcome (Win/Loss).

## Materials and Methods

Raw performance indicators from UFC competition were acquired upon formal request via FightMetric LLC; the official statistics and analytics provider to the organization (James et al., 2017b). Data from male bouts which occurred between 2000 and 2015 (*n* = 2,873) were screened for analysis. Only fights where a winner was decided (*n* = 2,831) were included, with “no contests” (*n* = 29) and “draws” (*n* = 13) removed from the analyses. The following 11 common performance indicators were recorded during each round: total strikes attempted, total strikes landed, significant strikes landed, significant strikes attempted, significant distance strikes landed, significant distance strikes attempted, significant clinch strikes landed, significant ground strikes landed, takedowns attempted, takedowns landed, and offensive passes (Table 1). A cumulative total, of each performance indicator for both fighters, was calculated across individual fights. Successful (landed) overall strikes and significant strikes were also expressed as a percentage of their respective attempts to produce two accuracy variables for inclusion in the analysis. A total 13 performance indicators were therefore considered.

**Table 1:** Performance indicators and associated definitions from mixed martial arts competition (James et al., 2017b).

**Performance indicator**	**Definition**
Total strikes attempted	All fully-committed attempts to strike an opponent
Total strikes landed	All fully-committed strike attempts that land with some measure of force
	All strikes landed as a percentage of those attempted
Significant strikes landed	All distance strikes that land with some measure of force, plus power strikes in the clinch and on the ground
Significant strikes attempted	All distance strikes attempted, plus power strikes attempted in the clinch and on the ground
	Significant strikes landed as a percentage of those attempted
Significant distance strikes landed	All strikes landed while the fighters are at distance
Significant distance strikes attempted	All strike attempts while the fighters are at distance
Significant clinch strikes landed	Power strikes landed while opponents are standing at close range
Significant ground strikes landed	Power strikes landed while opponents are on the ground
Takedowns landed	Successful grappling maneuvers that lead to control on the ground
Takedowns attempted	Grappling maneuvers intended to lead to control on the ground
Offensive passes	Positional improvements while on the ground in top position

*Power strikes: categorized by the provider using a series of cues including striking implement (i.e., leg vs. fist), velocity, hip, or torso rotation and follow through. At distance position: when fighters are not in sustained contact with each other. Clinch position: when the fighters are in sustained contact with each other. Ground position: when at least one fighter is considered grounded*.

Scatterplots used where relevant to visually describe bivariate relationships between the performance indicators, bout outcome, and bout duration. To provide a visual representation between fight outcome, year, and fight duration for each performance indicator, box, and whisker plots were employed. Using all performance indicators for winning bouts only, a multivariate analytical method, described below, was used to examine bout outcome over time (Zhu and Yu, 2009). Multivariate techniques differ to univariate approaches (for example, linear regression), by accounting for all performance indicators within a dataset along with both fixed and random factors. Thus, time and year can be considered within an analysis, rather than making inferences from models on specific indicators solely within years. The method used in the present study, non-metric multidimensional scaling (nMDS), has been utilized widely in ecology (Faith et al., 1987; Minchin, 1987) and recently, in sport, to examine the evolution of game style in Australian Football over a 15 year period (Woods et al., 2017a). In the present study, a matrix of dissimilarity scores, representing years and performance indicators from winning bouts, was created using the *metaMDS* function from the “vegan” package (Wagner, 2015) in R (R Statistical Package, 2009). Specifically, the Bray-Curtis dissimilarity feature was used in the nMDS to calculate the dissimilarity and temporal change of winning bouts over the 15-year period.

A propositional rule learner, the Repeated Incremental Pruning to Produce Error Reduction (RIPPER) (Cohen, 1995) was run to determine a set of rules in order to explain bout outcome (Win or Loss). This approach was chosen due to the incremental nature of the algorithm, by starting with less prevalent rules, then undertakes growing and pruning. The potential advantage of this approach, over decision trees for example, is performance with respect to overfitting, whereby decision trees likely work well on training but not validation or test sets. For example, in particle physics analysis (Britsch et al., 2009) of selecting signaling events, RIPPER outperformed other multivariate techniques. Similarly, in a business application of predicting a company's competitiveness (Wüthrich, 1997), rule-based approaches outperformed linear regression and decision tree techniques. The output of the RIPPER algorithm is also easy to understand and convey to a non-statistical audience, therefore making it practically useful and actionable information in a sport science setting. Recently, RIPPER was used to assess the effect of physiological and anthropometrical measures on draft selection in elite Australian Football (Robertson et al., 2015). In the present study, 13 performance indicators were considered for inclusion in the model, along with duration (min) and year. Only fights consisting of three rounds (15 min) or less were included. This analysis was run using the *JRip* function from the “RWeka” package. In order to reduce the potential of overfitting, the minimum number of instances was set to 75. A randomized 80:20 data split was used for model construction, with classification accuracy reported for Win and Loss outcomes on both training and test datasets. Splitting an entire dataset is common practice in machine learning, to evaluate model fit on unseen data.

## Results

**Figure 1:**
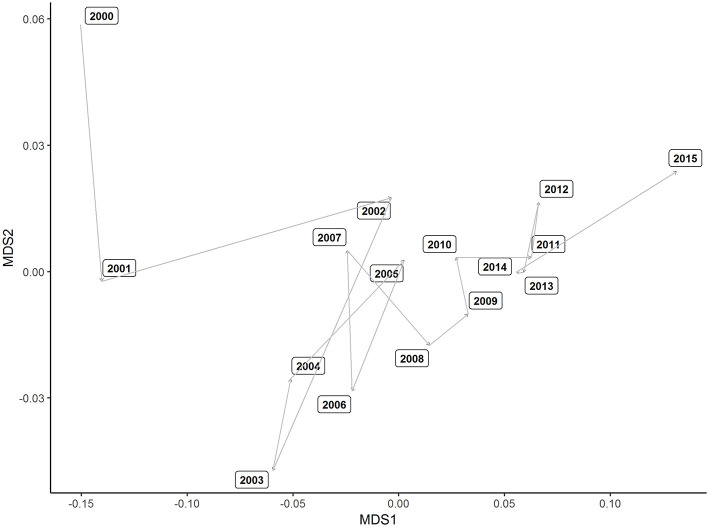
Ordination plot using nMDS of a dissimilarity matrix calculated from winning performance indicators by year. Larger spaces between year on the plot's ordination surface indicates greater dissimilarity between years.

**Figure 2:**
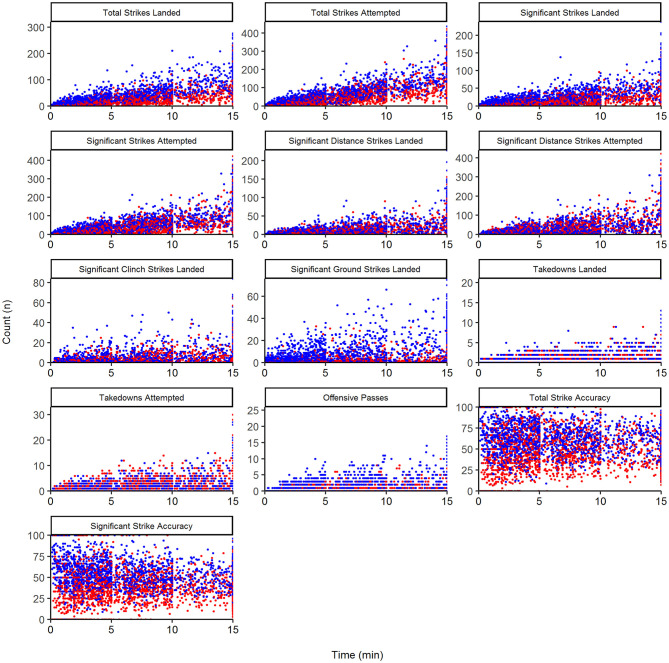
Scatterplot of raw performance indicators, as a function of bout outcome (win vs. loss) and duration (time). Red points, losing bouts; Blue points, winning bouts. Therefore, for any of the 13 performance indicators, a greater distinction in count number between red and blue dots at a given time point represents a greater difference between winners and losers.

**Figure 3:**
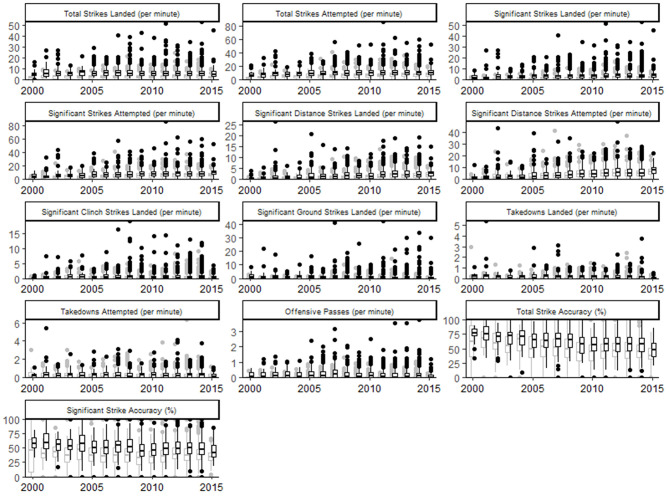
Box and whisker plots describing fight outcome, by year, expressed relative to fight duration for each performance indicator. Gray, losing outcome; Black, winning outcome.

The dissimilarity of multivariate, winning fight bouts was reached after 20 runs (stress = 0.04, maximum residual = 0.01). The dissimilarity plot demonstrates a clear shift in performance indicators used from 2000 to 2001 and 2002 (Figure 1). A small cluster of years 2003, 2004, and 2006 is then distinct before winning bout duration is clearly similar between 2005 and 2014 (Figure 1). In 2015, winning bout performance indicators appear clearly dissimilar to other years examined. Scatterplots, to visualize the univariate relationships between the performance indicators, bout outcome and bout duration are displayed in Figure 2. Figure 3 describes fight outcome, by year, expressed relative to fight duration for each performance indicator.

**Figure 4:**
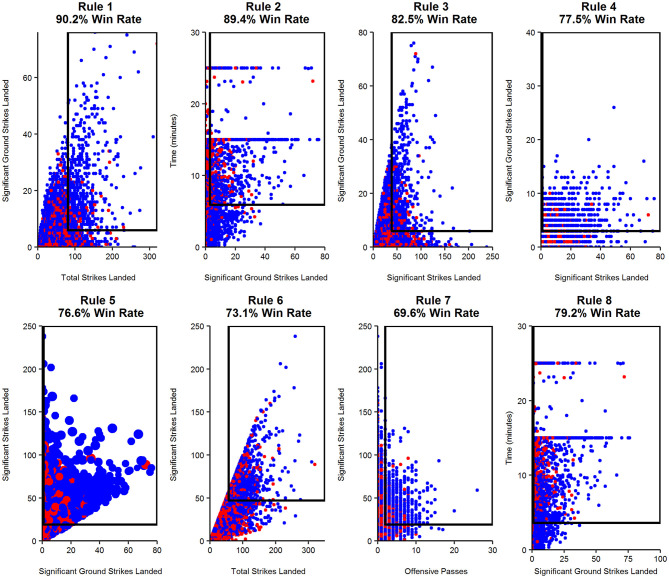
A visual representation of each rule and the resulting successful fight outcome, as a percentage. Red points, losing bouts; Blue points, winning bouts. Point size in Rule 5 indicate bout duration (minutes), with larger points a visual representation of 25-min bouts.

From the RIPPER model, eight rules were generated (Table 2). A visual of each rule is provided in Figure 4.

**Table 2:** Rules generated from the Repeated Incremental Pruning to Produce Error Reduction propositional rule learner across 2,831 Ultimate Fighting Championship Bouts between 2000 and 2015.

**Rule number**	**Rule description**	**Outcome (true positive/false positive)**
1	(Significant Ground Strikes Landed ≥ 6) and (Total Strikes Landed ≥ 81)	Win (559/61)
2	(Significant Ground Strikes Landed ≥ 3) and (Total Time ≥ 5.9)	Win (563/67)
3	(Significant Strikes Landed ≥ 39) and (Significant Ground Strikes Landed ≥ 6)	Win (207/44)
4	(Significant Ground Strikes Landed ≥1) and (Offensive Passes ≥ 3)	Win (414/120)
5	(Significant Ground Strikes Landed ≥ 1) and (Total Time ≤ 11.1) and (Significant Strikes Landed ≥ 19)	Win (242/74)
6	(Total Strikes Landed ≥ 57) and (Significant Strikes Landed ≥ 47)	Win (500/184)
7	(Offensive Passes ≥ 2) and (Significant Strikes Landed ≥ 19)	Win (103/45)
8	(Significant Ground Strikes Landed ≥ 1) and (Total Time ≤ 3.6)	Win (171/45)

*The rules are listed in order of prevalence in the dataset*.

For the training data set, fight outcome was classified at 77.0% accuracy (77.4% and 77.6% for Win and Loss, respectively). In testing, performance was slightly reduced to 75.2% overall (80.5% and 70.1% for Win and Loss, respectively). Further details relating to the overall performance of the model are shown in Table 3.

**Table 3:** Model accuracy statistics per fight outcome.

**Outcome**	**Recall**	**False positive rate**	**Precision**
Win	0.805	0.299	0.723
Loss	0.701	0.195	0.787

## Discussion

This study described changes in the nature of combat activity (as identified via key performance indicators) within the UFC between the inception of the Unified Rules of MMA in 2000 and 2015. Furthermore, the interaction between bout duration and performance indicator characteristics suggests that combat patterns change in accordance with the length of a bout.

Between 2000 and 2015, there was a considerable shift in the collective metrics describing a winning outcome (as represented by the nMDS, Figure 1), indicating a substantial change in the nature of competition in the UFC. However, a defined pattern or systematic progression throughout the period was not present and therefore, there was no clear presence of “eras.” These findings are in contrast to more established sports like Australian Football, where distinct eras in game-play have been reported (Woods et al., 2017a). Between 2000 and 2008, there were distinct and erratic shifts in performance indicator characteristics, particularly between 2000 and 2004, which may indicate experimental tactical strategies across the entire organization. These dramatic changes were possibly an attempt to capitalize on the broad sweeping rule changes brought about by the adoption of the Unified Rules of MMA by the UFC. Changes in 2000 included the removal of particular strikes and the inclusion of more well-defined weight classes (Smith, 2009). Consequently, there was a greater reliance on technical over physical factors, necessitating exploratory technical and tactical strategies in the years immediately following. Furthermore, the increased professionalism of the sport encouraged fighters to direct more resources to informed coaching practices (Birren and Schmitt, 2017) which, in turn, likely influenced combat activity.

A period of relative stabilization of combat patterns can be seen between 2008 and 2014 and likely reflects a degree of continuity in the technical nature of MMA competition. A notable finding is an increased rate of Significant Distance Strikes Attempted, and Landed, Per Minute, from the previous period (Figure 3). This reflects a shift in combat strategy from grappling emphasized tactics to a greater use of striking techniques at range. These striking attacks were historically seen as risky as it provided greater openings to counter attacks when compared to more conservative grappling techniques (Maher, 2009). Although a variety of factors may explain this finding, it is possible that more informed tactical behavior has allowed fighters to focus on developing striking capabilities that have ultimately influenced performance across the sport in its entirety. It is noteworthy that there appears to be another shift in performance indicator profiles between 2014 and 2015 which may suggest a new period of experimentation by athletes during competition.

Although there are substantial differences in many performance indicators, the difference between Win and Loss was generally unclear. This lends itself to the notion that not all performance indicators should be considered concomitantly, and their relevance is therefore heavily influenced by the combat situation (James et al., 2017b). The output from the RIPPER analysis overcomes this limitation by identifying combinations of specific performance indicators relating to fight outcome. This allows for concentration on a smaller number of performance indicators (in this case approximately one third of those included) and provides insight into how they specifically interact with one another, along with their level of interdependence. This is useful when considering human end users of the analysis output, as the limitations of humans in processing and interpreting excessive pieces of quantitative information is well-established (Miller, 1956). These data are in alignment with a previous investigation into MMA activity patterns using non-linear approaches (James et al., 2017b) and therefore adds further support to the advantages of such modeling techniques for performance indicator datasets.

The absence of “Year” in the generated rules is in agreement with the nMDS results, whereby a consistent and systematic evolution of combat activity as a function of year was not identified. In summary, the primary factors that determine victory were not influenced by the year in which a given bout occurred. This is similar to analysis of semi-professional netball competition (Bruce et al., 2018), whereby no evolutionary trend was evident over an 8 year period examined. Together with the present study, these results suggest that there is no characteristic “style of play” from year to year, in contrast with findings in Australian Rules football (Woods et al., 2017a) and rugby league (Woods et al., 2017b), whereby teams aim to typically replicate the performance strategy of a grand final winning team.

In contrast, bout duration is one of the few indicators represented in the RIPPER output, appearing in three of the eight rules (Figure 4). While Significant Ground Strikes Landed is highly influential regardless of bout duration, even a single execution of this metric (i.e., = >1) will result in a high likelihood of victory (77–79%) in fights of brief (<4 min) or long (<11 min) durations. Of particular relevance is the finding that bout duration interacts with Significant Ground Strikes Landed in each of the three rules in which it appears. Ground combat and successful striking indicators also feature in the rule approach, suggesting that offensive grappling and technical striking capabilities are predictive of MMA success. A similar finding was previously identified in a smaller sample (*n* = 234) of UFC bouts without considering the role of bout duration or year (James et al., 2017b), but nonetheless demonstrates that such techniques have remained important across the 15 years since 2000.

The relationship between bout duration and performance indicators characteristics is of particular interest due to the high unpredictability of fight length (James et al., 2016). Figure 2 indicates that while some performance indicators appear to change proportionally with time (e.g., a gradual increase in Total Strikes Attempted as time increases), there are several that contain different patterns. Notably, although there is a steady increase in Significant Distance Strikes Attempted over bout time, there is little change in the total amount that successfully land if a bout extends beyond 10 min. Accordingly, Significant Strike Accuracy is reduced over time, and the difference between winners and losers in this performance indicator becomes less distinct (represented by a reduction in accuracy by winners, and an increase by losers). Combined, these metrics indicate that the accumulated fatigue over the course of a bout has a considerable impact distance striking accuracy, and that this effect differs between winners and losers. Practitioners should therefore be aware of the impact of physical qualities (fitness) on combat activity (James et al., 2017a, 2018). Another interesting finding is that bouts that enter a 3rd round (i.e., >10 min) show a reduced number of Significant Ground Strikes Landed, despite Takedowns maintaining or increasing their frequency. This may be an indication of a conservative offensive grappling strategy where retention of a dominant position on the ground is prioritized over the delivery of strikes from that position during the final minutes of a bout.

These findings indicate a collective shift of combat activity within the UFC in the 15-year period since 2000. The identified changes are indicative of emerging technical and tactical and behavior as athletes and coaches attempt to adapt combat to determine effective strategies and tactics within a relatively modern sport. Although changes in performance indicator profiles have occurred between 2000 and 2015, the primary factors that determine victory have remained unaffected systematically by year.

From a practical perspective coaches and fighters can be confident that the factors currently influencing victory (i.e., offensive grappling and striking precision) will continue to be important. It is also necessary for practitioners to note that combat patterns change in bouts of longer duration. The reduction in Significant Ground Strikes Landed with no decrease in successful Takedowns by winners suggests that risk is minimized by the offensive fighter during ground combat (e.g., secure maintenance of top position rather than continued attacks from this position) as the bout draws to a close. This information can be considered by coaches when formulating both pre-determined game plans, and real-time tactical adjustments.

These findings should be interpreted within the context of the study's limitations. Firstly, the analysis did not consider the impact of weight classes. It is possible that the changes in combat patterns over time may not necessarily be similar across these different classes. Furthermore, this investigation is delimited to male bouts. Considering the recent increase in professionalization within the women's division, it would be of value to understand that development of combat behavior in recent years.

## Conclusion

This work describes a distinct shift (albeit without trend) in performance indicator characteristics across the entire UFC competition since the inception of the unified rules in 2000 until 2015. This is represented by erratic changes in skill profiles between 2000 and 2008, before relative stabilization to 2014. A notable factor influencing the development of combat in the UFC is the increasing use of distance striking techniques, and consequently, a more diverse combat style beyond the historically dominant grappling focused strategies. However, the key factors that determine victory in MMA (offensive grappling and precise striking) have remained unchanged over the investigated period. These findings also reveal changes in combat activity when bouts extend to longer durations. In these situations, winning fighters exhibit a reduction in striking accuracy toward the end of a bout, particularly in Significant Distance Strikes. Further, although still attaining considerably more Significant Ground Strikes Landed than losers, winning fighters show a trend to decrease this metric in longer fights, despite no reduction in Takedowns. Future work should look to incorporate weight divisions, fighting styles and males/females into the modeling approaches used to determine whether increased specificity can be obtained with respect to the generated findings. Further, time-series analysis of continuous or aggregate data, including performance indicators, may be beneficial to examine “eras” or changes in fight strategy over time. Recently, a time-series analysis approach was utilized to discover changes within Australian Football athlete physical output data, across a match stint or rotation (Corbett et al., 2019). Between six and eight dissimilar segments were discovered within a stint, meaning that specific training drills or rotation strategies could be employed, as a result of the specific change in match physical output over time. Future research could therefore apply a similar time-series approach to investigate how performance indicators change within a bout or year and across eras.

## Data Availability Statement

The datasets generated for this study will not be made publicly available as the dataset was acquired under individual agreement with FightMetric. This company is the data analytics service that compiled the dataset.

## Author Contributions

LJ, SR, and VK: conceptualization. SR: methodology. AS, SR, and LJ: formal analysis, interpretation, and writing—original draft preparation. LJ, SR, AS, and VK: writing—review and editing. LJ: project administration.

### Conflict of Interest

The authors declare that the research was conducted in the absence of any commercial or financial relationships that could be construed as a potential conflict of interest.
